# Victoria Hospital, Folkestone

**Published:** 1902-07-12

**Authors:** 


### The Institutional Workshop.

### VICTORIA HOSPITAL, FOLKESTONE.

This hospital, which was established in 1846 as a relatively small institution, was rebuilt in 1890 and enlarged by the addition of a new block of wards at the time of the Jubilee at a cost of about £7,700. The new wards are well constructed and attractive, and the lavatory and other fittings are of the best. Each ward contains 14 beds with central and open fireplaces without chimneys, and they present an air of great comfort to the eye. They are connected with the original building by a corridor, the bathrooms and kitchens being at one end of the wards and the lavatories and slop sinks at the other.

### Points for the Managers.

The experienced inspector on visiting this hospital is at once struck by the evidence there is of an absence of continuous supervision in regard to a number of relatively small matters which in the whole make for efficiency or the opposite. Outside the building, for example, it was noticeable that the areas had not been swept or dealt with for many months and probably for a longer period; that some of the gulley traps were full or nearly so, and that surrounding them were matters which could not have been found there under a system of proper inspection and flushing. We observed the same absence of attention to details and strict tidiness inside the hospital, especially in the basement and the larder. Upstairs in the wards we noticed that one of the children's wards, an unusually narrow one, contained too many beds, and that the temperature was far higher than was desirable or necessary. This may have been due to the fact that warmer weather has at length set in, and that a large fire was burning in the ward, which was certainly unnecessary on the day of our visit. We would recommend that two of the cots in the larger of the two wards devoted to children should be removed, as the necessary cubic and superficial space are lacking, and in their absence the retention of these cots is not justifiable in our opinion. We observed too that in some of the nurses' rooms new blinds are urgently needed, and indeed necessary, and we would recommend that most or all of these bedrooms should be redecorated.

### The Mortuary.

This building is situated at the bottom of the garden, well out of sight, and presents several features which are excellent. We observed, however, that there is no mortuary chapel where the friends can see their dead in decency, with all the surroundings which do so much to comfort the afflicted in such circumstances and to enhance the popularity of a hospital. At the present time we understand that the friends are shown into one of the chambers adjacent to the post-mortem room, each of which contains two large slate shelves on which the shells are placed. Such an arrangement is most undesirable, and should be discontinued, especially as there is ample space to make an excellent mortuary chapel at very little expense out of a room at the other end of the mortuary building, which at present contains nothing but a few linen coats for the doctors and some shelves for specimens. These specimens could be perfectly well placed in the post-mortem room and the shelves removed there, which would then leave a suitable room available for conversion into a mortuary chapel as we suggest. Having regard to the character of the institution, the beauty of its gardens and site, and the general excellence of the buildings we would urge the committee to at once take in hand this mortuary chapel. We cannot but believe that if the requirement is made known to the inhabitants of Folkestone, one or several of them will very gladly find the necessary money to have the chapel lined with tiles and converted into one of the most attractive features of the Victoria Hospital.

### Finance.

Knowing the large number of well-to-do people who live at Folkestone we were very much impressed with the statement that there are quite 800 of the principal residents occupying some of the best houses in the town and district who contribute nothing to the support of the hospital. We are further aware that Folkestone contains a large number of artisans, chiefly connected, we believe, with the building trade, which is in a flourishing condition, and it should be possible by organisation to considerably increase the contributions derived from this source. It it desirable for a small finance committee of business men to be appointed with authority to inquire into the expenditure and income accounts and to bring up a report embodying recommendations and a scheme for approaching the 800 non-subscribing but influential and prosperous residents. The same committee could establish a hospital week so as to promote an increase in the contributions from the artisan classes. Can there be any feeling at the moment that this hospital is extravagantly managed? We ask this question because we note that the cost per occupied bed, which was only £71 4s. 3d. in 1895, has increased to £89 18s. 2d. in 1901; the average daily number of in-patients in the hospital throughout 1895 was 28, and in 1901, 29·4. The average cost per patient per day in 1895 is given as 3s. 11d., and in 1901 as 4s. 11d. How is this increase in cost accounted for, and to what is it due? The average cost of an occupied bed in a hospital of this size should not exceed £70 per annum, and might be even less with improved efficiency under an active and intelligent system of management.

### Good Points.

As we have already said the fittings in the new wards are good; the store-room was a model of neatness and proved that the matron is well up to her work and has a good knowledge of housekeeping and arrangement. The grounds are very attractive, and must be a great boon to the patients in fine weather. The linen room was well stocked and in good order, but would be much improved if the cupboards containing the linen were to be properly ventilated, as they might easily be by the expenditure of a very small sum. In regard to the nursing, we understand that nearly the whole of it is entrusted to probationers, who pay fees during their first year. Having regard to the size and importance of the institution, it appeared to us from what we gathered that the proportion of probationers to trained and paid nurses was too large. The actual amount expended on nurses' wages was only £113 during last year, a sum which we regard as far too small for an institution containing rather more that 50 beds.

